# Preferred Features of Oral Treatments and Predictors of Non-Adherence: Two Web-Based Choice Experiments in Multiple Sclerosis Patients

**DOI:** 10.2196/ijmr.3776

**Published:** 2015-03-05

**Authors:** Paul Wicks, David Brandes, Jinhee Park, Dimitri Liakhovitski, Tatiana Koudinova, Rahul Sasane

**Affiliations:** ^1^PatientsLikeMeCambridge, MAUnited States; ^2^Hope Neurology / Hope MS CenterKnoxville, TNUnited States; ^3^Novartis Pharmaceuticals IncEast Hanover, NJUnited States; ^4^GfK Custom ResearchEast Hanover, NJUnited States

**Keywords:** multiple sclerosis, drug therapy, decision making, cross-sectional survey

## Abstract

**Background:**

Oral disease modifying therapies (DMTs) for multiple sclerosis (MS) differ in efficacy, tolerability, and safety.

**Objective:**

We sought to understand how these attributes impact patient preference and predicted DMT non-adherence among oral-naïve MS patients.

**Methods:**

Adult MS patients from the “PatientsLikeMe” Web-based health data-sharing platform completed a discrete choice exercise where they were asked to express their preference for one of three hypothetical oral DMTs, each with a certain combination of levels of tested attributes. Another Web-based exercise tested a number of possible drivers of non-adherence, mainly side effects. Data from an MS clinic were used to adjust for sample bias. Respondents’ preferences were analyzed using Hierarchical Bayesian estimation.

**Results:**

A total of 319 patients completed all questions. Most respondents were female (77.7%, 248/319) with mean age 48 years (SD 10). Liver toxicity was the attribute that emerged as the most important driver of patient preference (25.8%, relative importance out of 100%), followed by severe side effects (15.3%), delay to disability progression (10.7%), and common side effects (10.4%). The most important drivers of predicted non-adherence were frequency of daily dosing (17.4% out of 100%), hair thinning (14.8%), use during pregnancy (14.1%), severe side effects (13.8%), and diarrhea (13.0%).

**Conclusions:**

Understanding the important concerns expressed by patients may help health care providers to understand and educate their patients more completely about these concerns. This knowledge may therefore improve both choices of appropriate therapy and adherence to therapy over time.

## Introduction

Recent years have seen the introduction of a number of oral disease modifying therapies (DMTs) to the multiple sclerosis (MS) armamentarium, which supplements the earlier range of injectable and intravenous DMTs [1]. Adherence to injectable DMTs can be especially challenging for patients; although patients can often be resourceful in using coping strategies for injectable medications, reduced adherence remains an issue [2]. Three new oral DMTs have been approved for the treatment of MS: fingolimod (Gilenya, Novartis, FDA Orange Book approval date September 21, 2010), teriflunomide (Aubagio, Genzyme/Sanofi, FDA Orange Book approval date September 12, 2012), and dimethyl fumarate (Tecfidera, Biogen Idec, FDA Orange book approval date March 27, 2013). These choices change the landscape considerably. Each oral DMT occupies a unique niche in terms of its quantified ability to delay MS rate of progression, reduce frequency of relapses, alter lesion burden visible on magnetic resonance imaging (MRI), and contribute to side effects or serious adverse events. Physicians and patients must become increasingly involved in tradeoffs between efficacy, convenience, and safety in decisions and, interestingly, each approaches these factors from different perspectives [3].

One technique that helps examine the tradeoffs made by patients is “consider jointly” analysis (“conjoint” analysis). In this approach, participants are asked to indicate their preference from one of several discrete choices for a number of consecutive hypothetical product profiles that differ by varying levels of selected attributes. Such techniques have been used in studies of other chronic illnesses with complex decisions and tradeoffs to be made about treatments, with several studies in serious illness [4]. Because conjoint analysis takes multiple attributes into account simultaneously, conjoint studies allow researchers to create more complex models of decision-making than responses on simple rating scales. For example, in a sample of men with prostate cancer, a conjoint analysis study found that men were willing to trade off life expectancy to be relieved of certain side effects of treatment, and that preferences differed by age [5]. Similar work has also been applied to tradeoffs between different treatment characteristics in acne vulgaris [6] and pain control in osteoarthritis [7].

In MS, Johnson et al [8] used a Web-based conjoint analysis to study risk tolerance in a group of over 600 MS patients including those who had previously used natalizumab (which has been associated with an increased risk of progressive multifocal leukoencephalopathy/PML [9]). In showing patients different levels of efficacy and risk for hypothetical treatments, they found the most important attributes influencing patient preference to be the effect of a treatment to slow disease progression (27% out of a possible 100%), closely followed by risk of PML (23%), liver failure (20%), leukemia (18%), and reduction in the frequency of relapse (12%) [9]. The authors concluded that patients were willing to make tradeoffs of risk in exchange for improved DMT efficacy.

Given the recent availability of oral DMTs and the complex factors underlying decisions about selecting one, we sought to explore the relative preferences of a sample of oral-naïve MS patients (who have never taken any oral DMTs) with regards to salient oral DMT attributes. Our primary objective was to quantify and rank these attributes. Given that injectable DMTs have a variety of barriers to adherence that are rendered irrelevant by oral DMTs, we also sought to quantify and rank attributes that might affect the likelihood of non-adherence to the oral DMTs.

## Methods

### Recruitment

Methods are reported in accordance with the Checklist for Reporting Results of Internet Surveys (CHERRIES) [10]. Over a 10-day period in July 2013, we fielded a cross-sectional survey to a population of existing oral-naïve members from the “PatientsLikeMe” Web-based health-data sharing platform who reported a diagnosis of multiple sclerosis, were aged 18 years or over, and living in the United States. Patients reporting prior use of oral DMTs were excluded to maintain oral naivety and infrequently prescribed DMTs (such as Extavia) were excluded from the study to avoid cells with small Ns for post-hoc analyses.

Members who sign up to PatientsLikeMe do so under the terms of use, which make clear they could be contacted for research; additional informed consent was collected for this voluntary study. Potential participants were selected on the basis of previously submitted profile data and were contacted via email. Participants were informed of the study sponsor, the objectives of the study, that it would take approximately 15 minutes to complete, and that a US $25 cash card incentive would be provided to those who completed the study. Consenting to the study took patients to a Web-based survey tool hosted by GfK Custom Research. To avoid missing or spurious data, all questions were mandatory to complete the survey, participants could not revise earlier answers, and unique URLs were used to avoid the risk of multiple completions or spurious data entries. Institutional review board (IRB) approval was granted from Western IRB.

### Survey Development

Survey measures included basic demographics, MS DMT history, patient-reported disease severity (MS Rating Scale revised, MSRS-R) [11], the MS Treatment Adherence Questionnaire (MSTAQ) [2], and the Beliefs about Medicines Questionnaire (BMQ) [12].

### Treatment Characteristics Preference Exercise (CA1)

**Figure 1:**
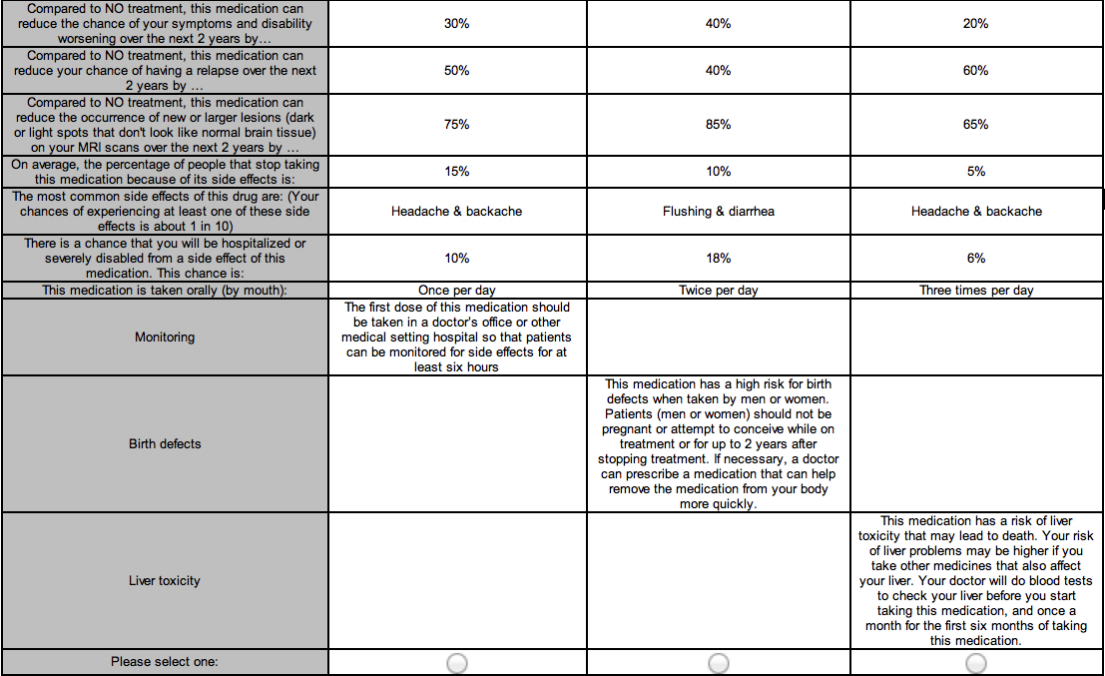
Example screenshot from Conjoint Analysis Exercise 1 (CA1) – Participants were asked “Of these three products, which would you be most likely to ask your physician to prescribe to you if these were the only options available?".

**Figure 2:**
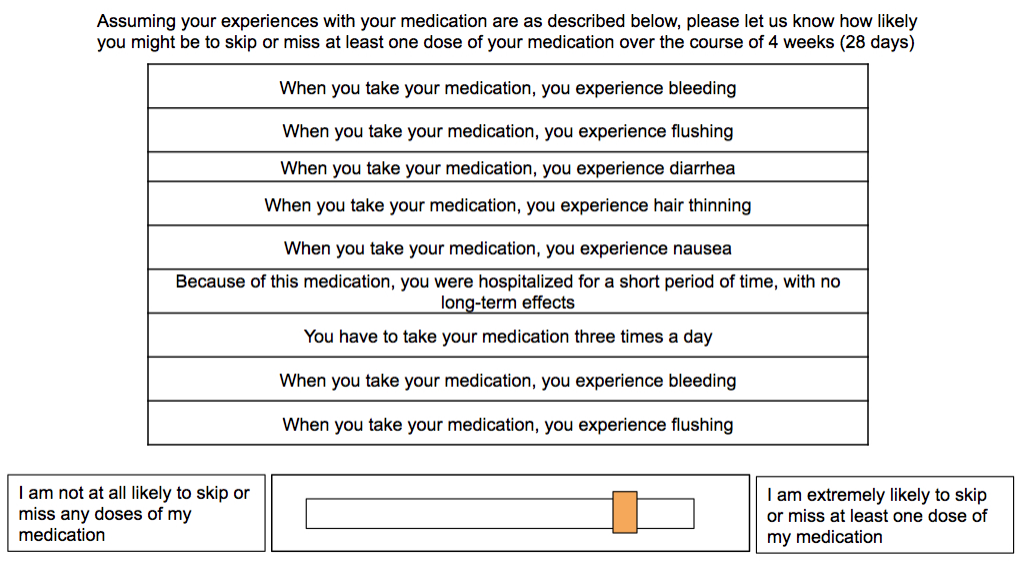
Example screenshot from Conjoint Analysis Exercise 2 (CA2) – Participants moved the yellow sliders between the two extreme values.

We developed two conjoint analysis tasks in accordance with International Society For Pharmacoeconomics and Outcomes Research (ISPOR) guidance on use of conjoint analysis [11]. The first exercise (CA1) asked oral-naïve MS patients to repeatedly choose one of three hypothetical oral DMTs, each with a certain combination of levels of tested attributes (shown in Table 1). Example screenshots of the tasks are shown in Figures 1 and 2.

**Table 1:** Parameters and values for Conjoint Analysis Exercise 1 (CA1) ranked by order of importance to oral-naïve MS patients (n=319).

Attribute	Description	Value Level 1	Value Level 2	Value Level 3	Value Level 4	Relative importance (out of 100%)
Liver toxicity	This medication has a risk of liver toxicity that may lead to death. Your risk of liver problems may be higher if you take other medicines that also affect your liver. Your doctor will do blood tests to check your liver before you start taking this medication, and once a month for the first six months of taking this medication.	Yes	No	−	−	25.8%
Severe side effects	There is a _____ chance that you will be hospitalized or severely disabled from a side effect of this medication.	6%	10%	14%	18%	15.3%
Delay the progression of disability	Compared to no treatment, this medication can reduce the chance of your symptoms and disability worsening over the next 2 years by…	20%	30%	40%	−	10.7%
Common side effects	The most common side effects of this drug are … Your chances of experiencing at least one of these side effects is about 1 in 10.	Headache, backache	Flushing, diarrhea	Hair thinning, nausea	−	10.4%
Frequency of administration	This medication is taken orally (by mouth)…	Once per day	Twice per day	Three times per day	−	9.5%
Reduce frequency of relapses	Compared to no treatment, this medication can reduce your chance of having a relapse over the next 2 years by…	30%	40%	50%	60%	8.7%
Reduce changes on MRI	Compared to no treatment, this medication can reduce the occurrence of new or larger lesions (dark or light spots that don't look like normal brain tissue) on your MRI scans over the next 2 years by…	65%	75%	85%	−	6.7%
First dose monitoring	The first dose of this medication should be taken in a doctor’s office or other medical setting hospital so that patients can be monitored for side effects for at least six hours.	Yes	No	−	−	4.6%
Tolerability	On average, _____ of people stop taking this medication because of its side effects.	5%	10%	15%	−	4.5%
Birth defects	This medication has a high risk for birth defects when taken by men or women. Patients (men or women) should not be pregnant or attempt to conceive while on treatment or for up to 2 years after stopping treatment. If necessary, a doctor can prescribe a medication that can help remove the medication from your body more quickly.	Yes	No	−	−	3.8%

### Non-Adherence Exercise (CA2)

The second exercise (CA2) showed patients just one hypothetical oral DMT but with varying levels of the values shown in Table 2 and a response task of a visual analogue scale they used to indicate how likely they might be to miss at least one dose of medication over the course of 4 weeks (28 days), with “I am not at all likely to skip or miss any doses of my medication” at one end and “I am extremely likely to skip or miss at least one dose of my medication” on the other. Both sets of attributes were identified through review of the clinical trial literature by two independent raters and consultation with a clinical expert (DB).

**Table 2:** Parameters and values for Conjoint Analysis Exercise 2 (CA2) ranked by order of importance to oral-naïve MS patients (n=319).

Attribute	Value Level 1	Value Level 2	Value Level 3	Relative importance (out of 100%)
Frequency of administration	Once per day	Twice per day	Three times per day	17.4%
Side effects − hair thinning	Yes	No	−	14.8%
Pregnancy − you or your partner become pregnant while taking your medication	Yes	No	−	14.1%
Severe side effects − hospitalized for a short period of time, with no long-term effects	Yes	No	−	13.8%
Side effects − diarrhea	Yes	No	−	13.0%
Side effects − nausea	Yes	No	−	10.7%
Side effects − backache	Yes	No	−	8.8%
Side effects − headache	Yes	No	−	4.5%
Side effects − flushing	Yes	No	−	3.0%

### Statistical Analysis

To help overcome the biases of a Web-based sample of patients from the PatientsLikeMe Web-based data platform, we used sample weights to adjust the proportions of the sample for groups using benchmarks from the Partners Northeast MS Center in the United States [13]. A SAS iterative proportional fitting macro created the weights by adjusting all target variables simultaneously. Extreme weights were then trimmed to reduce the influence of extreme outliers in the weighted results and improve weighting efficiency. Trimmed weights are shown in Table 3 (weighting was associated with a design effect of 1.27, or 79% weighting efficiency).

Data were analyzed using hierarchical Bayesian estimation. We estimated hierarchical Bayesian models in Sawtooth Software (Orme, USA) that uses a specific Monte Carlo Markov Chain Algorithm called the “Metropolis Hastings Algorithm”. At the end of the estimation, each level of each attribute is assigned a numeric value (“part worth” or “utility”) that reflects how much this level is valued by the respondent. Relative importance of the attributes derives from conjoint analysis and is based on the utilities. The attribute importance values add up to 100% for each conjoint analysis exercise. For each attribute, a difference between the highest and the lowest utility is calculated, and the relative importance is obtained by dividing that difference by the sum of the differences for all attributes. The target sample size (N=300) was determined using a power analysis assuming three comparable products for each decision task, a 6% margin of error, and a desired 95% confidence interval. Most published conjoint analysis studies have a sample size between 100 and 300 respondents, and our proposed sample size is consistent with guidance in the methodology literature [4].

## Results

From 1790 invited MS patients, 327 completed all questions. During data cleaning, responses from 8 patients were not analyzed (2 duplicates, 2 violated inclusion criteria, 4 for “straight-lining” answers), leaving a total of 319 (17.8% response rate, 319/1790). Most respondents were female (77.7%, 248/319) with mean age 48 years (SD 10) and a 10-year self-reported history of MS with 70.8% (226/319) reporting a diagnosis of relapsing remitting MS. Table 3 shows the raw unweighted sample demographics as well as the transformed sample following weighting to more closely resemble a representative MS population. All conjoint analysis data referred to from this point comes from the weighted sample used for analysis.

The most frequently impaired aspect of function on the MSRS-R was walking, with 53.6% (171/319) of patients moderately-severely impacted on this item, followed by 36.4% (116/319) experiencing sensory issues, 26.0% (83/319) cognitive issues, and 27.0% (87/319) bowel or bladder disturbance.

Most patients (61.0%, 195/319) were taking a DMT at the time of survey, with the most frequent being glatiramer acetate (29.5%, 94/319, Copaxone, subcutaneous daily injection, TEVA) followed by natalizumab (18.8%, 60/319, Tysabri, monthly intravenous transfusion, Biogen Idec), interferon beta-1a (9.1%, 29/319, Avonex, weekly intramuscular injection, Biogen Idec), and interferon beta-1b (8.8%, 28/319, Rebif, 3x weekly subcutaneous injection, EMD Serono). Approximately two-thirds (61.1%, 195/319) were taking a DMT at the time of survey, slightly higher than the Sonya Slifka longitudinal study, which found that 50.0% of patients were “currently” (in 2000-2001) using a DMT but similar to the reported 62.2% that had taken a DMT at some point in their disease [14].

The BMQ showed that overall, most patients (67.1%, 214/319) worried about long-term effects of their DMTs, with a higher proportion endorsing this sentiment among the patients who had missed at least one dose in the past 28 days (77.6% agreed or strongly agreed, 52/67, compared to those that hadn’t (57.8%, 74/128, χ^2^_195_= 9.780, *P*=.04).

Among the 195 patients using a DMT, most patients self-injected their DMT (69.2%, 135/195), with 22.1% (43/195) reporting that someone else helped them with the injection most or all of the time, and 8.7% (17/195) only half of injections or just a few times. Among the 156 patients who reported using an injectable DMT, 59.6% (93/156) used an auto-injector exclusively, 32.1% (50/156) injected manually, and 6.7% (13/195) used a mixture of both. Overall, ease of use with current treatment was relatively high, with 89.7% (175/195) of DMT users saying their treatment was either “easy to use” or only “a little hard” to use. Similarly, satisfaction was quite high, with most users reporting “moderately”, “very”, or “completely” satisfied (88.7%, 173/195) and only 11.3% reporting they were either “a little satisfied” or “not satisfied at all” (11.3%, 22/195).

About a third of DMT users (34.3%, 67/195) reported missing at least one dose in the previous 28 days, with the most common reasons being “did not feel like taking my medication” (35.8%, 24/67 reported as a “moderately” or “extremely” important factor), “memory problems” (26.9%, 18/67), and “tired of taking my medication” (28.4%, 19/67). Patients who reported missing a dose of their medication in the past 28 days were not significantly different than those who did not on their MSRS-R outcome (*t*_193_=1.730, *P*=.09).

**Table 3:** Sample demographics before and after weighting (n=319).^a^

		Unweighted sample frequencies (%)	Benchmark (Partners MS Center)	Weighted sample frequencies (%)
**Gender**				
	Female	248 (77.7%)	74.8%	238 (74.6%)
	Male	71 (22.3%)	25.2%	82 (25.7%)
**Age, years**				
	18-38	36 (11.2%)	25.0%	76 (23.8%)
	39-46	59 (18.5%)	25.0%	79 (24.7%)
	47-62	186 (58.3%)	40.0%	132 (41.3%)
	63+	38 (11.9%)	10.0%	33 (9.7%)
**Race**				
	White	283 (88.7%)	92.4%	292 (91.5%)
	Black	18 (5.6%)	4.6%	17 (5.3%)
	Other	18 (5.6%)	3.0%	10 (3.1%)
**MS subtype**				
	Relapsing-Remitting	240 (75.2%)	70.2%	225 (70.5%)
	Primary Progressive	25 (7.8%)	6.2%	18 (5.6%)
	Secondary Progressive	47 (14.7%)	22.6%	72 (22.6%)
	Progressive Relapsing	7 (2.1%)	1.0%	4 (1.2%)
**Highest education**				
	High school graduate or less	45 (14.1%)	19.4%	62 (19.4%)
	Some college	115 (36.1%)	54.8%	171 (53.6%)
	College graduate or more	159 (49.8%)	25.8%	86 (26.9%)

^a^Percentages may not add up to 100% due to rounding.

### Treatment Characteristics Preference Exercise (CA1)

Results from the CA1 preference exercise showed that potential for liver toxicity was the most important factor (Table 1, 25.8% as a measure of relative importance out of 100%) in hypothetical DMT selection followed by severe side effects (15.3%), delay to progression of disability (10.7%), common side effects (10.4%), and mode of administration (9.5%). Reducing the frequency of relapses (8.7%) and reducing changes on MRI (6.7%) were less important in driving preferences, as were requirement for a first-dose monitoring period (4.6%), tolerability (4.5%), and risk of birth defects (3.8%).

### Non-Adherence Exercise (CA2)

The CA2 non-adherence exercise (Table 2) found that the most important determinant of self-reported non-adherence to a hypothetical DMT was frequency of daily administration (17.4%), hair thinning (14.8%), becoming pregnant (14.1%), severe side effects (13.8%), and diarrhea (13.0%). Nausea (10.7%), backache (8.8%), headache (4.5%), and flushing (3.0%) emerged as less important drivers of non-adherence. A tradeoff simulator was built for CA2, which allows prediction of non-adherence for any combination of the relevant values of these parameters. For instance, in the worst-case scenario of a thrice-daily dosing of an oral DMT that causes hair thinning, has severe side effects, diarrhea, nausea, backache, headache, and flushing, the model predicted a 78% likelihood of missing at least one dose over the course of 4 weeks for an average patient. In the best-case scenario, a DMT with none of the tested side effects and once-daily dosing, the model predicted 15% likelihood of at least one missed dose. In simulations, the frequency of daily dosing had the largest incremental impact on adherence.

## Discussion

### Principal Findings

In this Web-based survey of oral DMT naïve patients, we found that liver toxicity, severe side effects, and common side effects were the most salient attributes driving DMT preference, with efficacy, frequency of dosing, and first dose monitoring less important. However, we also found that the frequency of dosing and specific side effects, such as hair thinning, might have an influence on patients’ predicted non-adherence to taking an oral DMT.

The landscape of MS is currently undergoing a transformation from self-injectable and intravenous DMTs to a wider range of delivery routes including oral agents [15]. Our findings suggest the primacy of serious adverse events like liver toxicity may be major drivers of patient preference. All three of the recently approved oral DMTs have some kind of hepatotoxic profile identified in their phase III trials, such as elevated alanine aminotransferase tests three times the normal range in 6-7% of patients taking dimethyl fumarate (with no reported hepatic failure) [16,17], 7-19% for fingolimod [18,19], and 7-12% for teriflunomide [20,21]. However, in this last case due to the known properties of leflunomide (which metabolizes to teriflunomide) in rheumatoid arthritis, the drug was issued with an FDA “black box” warning [22] for severe liver injury including fatal liver failure, requiring liver monitoring at least monthly for 6 months after treatment initiation. Patient concern over a similar risk appeared to be the major driver of patient preference in our hypothetical conjoint analysis and patients actually prescribed the drug might benefit from extra assurance that monitoring should identify any issues that arise.

Our results contrast with those of Johnson et al, who found a stronger preference for slowing disability (27%) than avoiding side effects, although the serious adverse event PML (23%) was a frequently endorsed driver of preference, as was the potential for liver failure (20%) [8]. The different findings between our two studies may reflect the different sample chosen, as at least 42% of Johnson et al’s sample had already taken natalizumab (Tysabri) at some point, which may reflect a higher risk tolerance. By contrast, only 21% of our sample were taking Tysabri, and about a third were not taking any form of DMT. Although speculative, it is also possible that reports of PML in the MS community have drawn attention to the fact that even rare adverse events can occur with serious consequences [23].

In the current study, patients expressed greater preference for product profiles with fewer serious side effects and fewer common side effects relative to those with higher levels of efficacy. The clinical trial literature suggests that serious adverse events were reported among 17-18% of patients taking dimethyl fumarate in trials [16,17], followed by 14-16% over 12 weeks [20] taking teriflunomide (29-36% over long term use [21]), with the fewest among patients taking fingolimod (7-10%) [18,19]. However, it is worth nothing that oral medications do not carry the injection-related profile of side effects such as injection site pain or erythema [17].

In agreement with Johnson et al, we found a higher preference for delaying the progression of disability over reducing the frequency of relapses. Establishing the former in typical clinical trials is much harder than the latter, requiring longer and larger studies. It may be that patients better understand the concept of progressive disability than they do of “relapses”, which are highly unpredictable and may be complex to disentangle from disability progression or pseudo-exacerbations.

As a complex condition involving many tradeoffs, there is increasing interest in the use of conjoint analysis techniques in various aspects of decision-making in MS and supporting MS patients to make better-informed decisions based on their personal treatment preferences [24]. Shingler et al describe the use of conjoint techniques to identify patient preferences for characteristics of self-injection devices [25] and found that a treatment’s efficacy mattered more to patients than ease of use to administer it, with technological features like medication reminders having relatively low importance. Over half of our sample were using an auto-injector for their DMT and reporting a good level of satisfaction, begging the question of whether patients with a relatively high level of satisfaction will appreciate as much of a difference between a self-injected DMT and an oral as earlier cohorts of patients who did not have the benefit of auto-injectors.

Medication non-adherence is a known issue in MS and a variety of solutions has been proposed to study this important issue. The largest (N=2648) and most rigorous study in this disease, the Global Adherence Project (GAP), found that 25% of patients were non-adherent to therapy, with memory being a major issue [26]. Although studies of injectable DMTs have found a number of issues related to route of administration or site injection reactions, it is unlikely that memory issues or treatment fatigue will be addressed solely by a move to oral DMT therapy. Although greater convenience would be anticipated, it is worth noting that many MS patients are already reporting a high level of satisfaction particularly due to the use of auto-injectors. One possible downside to oral DMTs is the absence of adherence-tracking technology that can be built into auto-injector devices, although systems such as the Proteus Raisin System might address such challenges in future [27].

### Limitations

This study had several limitations, including the examination of only hypothetical product profiles in a cross-sectional manner and what patients said they would choose in an artificial setting rather than the behavior they would actually exhibit. However, conjoint analysis may be considered ecologically valid because individuals are used to making decisions from among multiple varying choices on a daily basis [28]. In terms of study design, all conjoint studies suffer from a conceptual bias in that the questions they seek to address naturally constrain patient choice in a way that may not reflect the real world. For example, in the current study we asked patients to select between one of three oral MS DMTs, when they might have preferred self-injectable DMTs or second-line DMTs such as natalizumab—patients face a wide array of potential DMT options [1]. Another conjoint analysis limitation is accurately conveying clinical endpoints and the concept of risk such as percentage changes; we attempted to use endpoints that were commonly used in the MS community (and vetted these with a clinician) but it is certainly feasible that cognitive biases or comprehension issues limited full understanding.

As an online community, PatientsLikeMe users exhibit biases relative to other clinical samples such as MS patients at a specialist MS center [13] including being younger or more likely to be female; we sought to address this through sample weighting. While weighting reduces the bias in results, it does increase the variance of the results, resulting in a decreased statistical sensitivity to detect differences between groups. Our sample contained a relatively high proportion of Copaxone users, which might affect the results by including a larger set of patients using a lower-risk drug. However, inspection of the conjoint analysis results suggest that there were no major differences between the preferences of current Copaxone users relative to those who have never used it, with differences in CA1 importance levels of just 1% or less. The sample also included a relatively high proportion of patients not taking any DMT. It is unclear why these participants were not taking a DMT but we felt it was important to include the results as they represent a proportion of the population that might one day stand to benefit from a DMT if their expectations can be met. Given the heterogeneity of experiences with medication adherence, defining “non-adherence” as a single missed dose in the past 28 days is overly simplistic, but as the number in this group was relatively low, any further subdivision would have lead to very low N’s for statistical analysis.

Given the self-reported nature of the site, we have no evidence to confirm that members saying they have been diagnosed with MS have actually done so, nor that this diagnosis was accurate. Research is underway to more systematically address this limitation, but we intentionally targeted for recruitment those members who had been active on the site in the preceding 120 days rather than recruiting a new sample through advertising, so the likelihood that a given member would have signed up more than three months previously and maintained an active but fake account just on the chance of later gaining a survey incentive seems low. We also cleaned the dataset for evidence of straight-lining or duplicate entry. It is conceivable that a subset of users might have gone on to have their diagnosis changed to another condition; however, these limitations are shared in common with many other studies that use mailing lists or Web-based recruitment techniques and held as a common caveat. In summary, we believe that all study methodologies have their own set of limitations but that Web-based techniques have the advantage of adapting to address these through iterative software upgrades.

Future areas of research might include studying how patients starting an oral DMT report making that decision and what their medication adherence is like long-term. There is an inherent assumption that moving from injectable to oral DMTs should produce improved adherence but this is yet to be tested rigorously.

In attempting to select the best of these therapeutic options for each patient, a balance must be struck of efficacy, safety, tolerability, adherence, potential need for monitoring, and cost effectiveness [29]. It is possible that the use of decision aids that personally tailor an individual patient’s attitudes to risk and lifestyle preferences, supported by quantitative data abstracted from the clinical literature, could prove a useful tool.

### Conclusions

Oral-naïve MS patients identified liver toxicity and serious side effects as the most significant determinants of DMT selection while high frequency of daily dosing and certain side effects appear to be the most important barriers to DMT adherence. The use of conjoint analysis could be helpful in the development of new decision aids to help patients and clinicians navigate their many choices of DMT.

## Abbreviations

BMQ: Beliefs about Medicines Questionnaire
CA: conjoint analysis
DMT: disease-modifying therapy
FDA: Food and Drug Administration
MRI: magnetic resonance imaging
MS: multiple sclerosis
MSRS: MS Rating Scale revised
MSTAQ: MS Treatment Adherence Questionnaire
PML: progressive multifocal leukoencephalopathy
